# The Chromaverse Is Colored by Triplexes Formed Through the Interactions of Noncoding RNAs with HNPRNPU, TP53, AGO, REL Proteins, Intrinsically-Disordered Regions, and Flipons

**DOI:** 10.3390/ijms27031482

**Published:** 2026-02-02

**Authors:** Alan Herbert

**Affiliations:** Discovery, InsideOutBio, Charlestown, MA 02129, USA; alan.herbert@insideoutbio.com

**Keywords:** triplexes, flipons, p53, AGO, PIWI, NFKB, RAD51, histone, DNA, methylation, SATB1, intrinsically disordered regions

## Abstract

Triplexes (TRX) are a class of flipons that can form due to the interaction of RNA with B-DNA. While many proteins have been proposed to bind triplexes, structural models of these interactions do not exist. Here, I present AlphaFold V3 (AF3) models that reveal interactions between the high-mobility group protein B1 (HMGB1), HNRNPU (SAF-A), TP53, ARGONAUTE (AGO), and REL domain proteins. The TRXs result from the sequence-specific docking of RNAs to DNA via Hoogsteen base pairing. The RNA and DNA strands in apolar TRX are oriented in the opposite 5′ to 3′ direction, while copolar TRX have RNA and DNA strands pointing in the same 5′ to 3′ direction. TRXs can incorporate different RNA classes, including long noncoding RNAs (lncRNAs), short RNAs, such as miRNAs, piRNAs, and tRNAs, nascent RNA fragments, and non-canonical base triplets. Many pathways regulated by TRX formation have evolved to constrain retroelements (EREs), which are both an existential threat to the host and a source of genotypic variation. TRXs help set the boundaries of active chromatin, repressing the expression of most EREs, while depending on other flipons to modulate cellular programs. The TRXs help nucleate folding of intrinsically disordered proteins.

## 1. Introduction

To survive and replicate, organisms must defend against many challenges. They must adapt to overcome not only current threats but also future ones. Replicants that are embedded within the host genome are particularly hard to defeat. Examples include retroelements that copy and paste themselves from one site to another, and viruses that rewire the cell. Yet at some point, the host must also protect the host genome if it is to persist. Many of these mechanisms trace back to the first known eukaryotic progenitors. They also reflect the evolution of regulatory schemes, starting with those that relied on nucleic acid structure and then progressing to base-specific interactions mediated by both RNAs and proteins.

The structure-based schemes depend on sequences that adopt alternative conformations. These sequences are called flipons [1]. They often consist of repeat-sequence motifs that can form distinct folds, each one representing a different energy minimum in the folding landscape. The flip from the Watson–Crick B-DNA to the left-handed Z-DNA duplex is favored by an alternating purine/pyrimidine motif and involves the inversion of base pairs in a cooperative transition. Formation of the 4-stranded G-quadruplex (GQ) structure occurs in sequences with a guanosine-repeat motif and results in a stable structure. The transition between Z- and G-flipon structures requires energy. The distinct surfaces of B-DNA, Z-DNA, and GQ enable interactions with structure-specific partners. It is likely that early in evolution, these structures served as scaffolds for various chemistries that enabled their self-replication. The entities involved have been called “tinkers”. It is thought that they consist of nucleic acid codon repeats that bind the dipeptides they specify in the modern-day genetic code. The nucleic acid and peptide polymers promote the synthesis of the other using bound metals as catalysts [2]. Later, sequence-specific interactions catalyzed the synthesis of the polymers and the elaboration of other structures. Eventually, proteins subsumed some of these functions.

Such flipon-dependent mechanisms have been considered non-canonical, mainly because they were not observed in the prokaryotes studied by the first generation of molecular biologists. Instead, transcription in these organisms was modulated by base-specific, high-affinity proteins. In contrast, flipons are frequent in vertebrate genomes, with many of the interactions that regulate transcription only occurring transiently [3]. The complexes they seed vary dynamically, rather than through a pre-specified lock-and-key mechanism. The particular complex formed depends on the flipon conformation, the proteins expressed in a cell, their availability, and their modifications [4]. The arrangement generates immense phenotypic variability without requiring a vast array of base-specific B-DNA-binding transcription factors, as is currently proposed [5]. Indeed, the genome consists of “HOT” loci that bind many transcription factors lacking a canonical binding motif in the region [6,7]. The ability of TFs to bind both B-DNA and GQs offered a resolution to this HOT dilemma [8].

This paper will explore how endogenous retroelements (EREs) shaped these very different evolutionary outcomes. The paper has seven distinct parts. The first part will focus on defending against EREs (Section 1.1). Two different strategies will be discussed: those based on alternative nucleic acid structures encoded by flipons and others based on ERE-specific sequences. The first scheme eliminates the need for pathways that amplify RNAs targeting EREs by RNA-dependent polymerases (RdPs). These RdPs play a key role in RNA interference (RNAi) pathways in organisms like *Caenorhabditis elegans*. The second part (Section 1.2) will discuss how ERE sequences have been exapted to play critical regulatory roles in animal genomes. These outcomes also depend on alternative nucleic acid structures encoded by flipons, and not solely on sequence-specific protein binding motifs. The third part (Section 1.3, Section 2.1, Section 2.2 and Section 2.3) will focus on the role of ERE transcripts in modulating host genes. These RNAs are involved in establishing nuclear structure and seeding transactional complexes that vary by context. Many of these RNAs seed distinct chromatin structures by forming a three-strand triplex (TRX) with host duplex DNA. The multiplicity of chromatin states produced is captured by the term ‘chromaverse’. Only one of the many possible chromatin states is present in each cell [9]. The fourth part (Section 2.4) focuses on proteins that recognize these TRXs. The analysis is based on predictive modeling using AlphaFold V3 (AF3) and is motivated by experimental findings that the proteins analyzed engage with TRXs. Part 5 (Section 2.5) models proteins that modulate TRX formation, and Part 6 (Section 2.6) examines the chromatin complexes seeded by TRXs, and the induced folding of proteins with intrinsically disordered regions (IDRs). Part 7 (Section 3) relates these findings to genetic encoding by flipons, offering a highly dynamic perspective of the genome. This work builds on a previous manuscript that examines the history and physical chemistry of TRXs and summarizes experimental evidence for their formation in cells [9].

### 1.1. Protecting the Genome Against Invasive Replicants

Every genome must defend against invasive pathogens. Different species have met this challenge in various ways. One approach involves producing pathogen-specific guide RNA (gRNA) sequences that direct cellular nucleases to attack the invader. Examples include the CRISPR, FANZOR, and retron systems of bacteria [10,11,12]. Some metazoans, such as *Caenorhabditis elegans*, have exploited the RNA interference system. They deploy host-encoded RNA-dependent RNA polymerases (RDRPs) to amplify pathogen-specific guide RNAs (gRNA) that engage AGO proteins with their target [13].

#### 1.1.1. PIWI and piRNAs

A different solution is required when genomes incorporate a high percentage of EREs capable of self-replication [14,15,16]. These elements utilize a copy-and-paste mechanism to colonize the host genome. They depend on an ERE encoded reverse transcriptases. Any host-encoded RDRP would enhance the ERE attack. This challenge can be overcome by generating gRNAs in different ways. One such system utilizes piRNAs to guide the AGO-related PIWI family of effectors to their targets. The piRNAs are produced either by “ping-pong” amplification (PPA) or by progressive production of piRNAs (PPPi) from ERE-derived transcripts [17]. PPA is initiated by the cleavage of overlapping sense and antisense RNAs containing pathogen sequences that are produced from clustered arrays of ERE within the host genome. The transcripts are made by the host DNA-dependent RNA polymerase (DdRp). The piRNAs generated from the sense strand drive the production of a piRNA from an antisense transcript. The subsequent amplification of either sense or antisense piRNAs depends on their match to ERE transcripts produced from other genomic locations. PPA is implemented in flies and vertebrates through the segregation of germ-line tissues. PPPi, in contrast, relies on reiterative slicing of an ERE transcript. Cleavage commences at the 5′ end of the RNA and continues for several cycles. PPPi is more common in vertebrates than PPA [17]. It is deployed during spermatogenesis and in the early stages of embryonic development. In both situations, chromatin decompaction leads to widespread bidirectional transcription of the genome. The piRNAs transmitted to embryos by both oocytes and sperm also help reestablish ERE suppression. In flies, a mismatch between the piRNAs inherited from the mother and the EREs from the father can lead to hybrid dysgenesis [18].

#### 1.1.2. ADAR, ZBP1, and ZNAs

Another scheme also protects tetrapods against EREs. The focus here is on humans, where over 50% of the genome is derived from retroelements, with the exact percentage depending on how each ERE family member is defined (e.g., whether the sequence is full-length, how many sequence variants are tolerated, etc.). This alternative system of protection is completely introspective and relies on host-encoded transcripts rather than ERE-specific guides. Instead, the alternative nucleic acid structures (ANS) are formed by EREs, rather than their sequence. ANS such as Z-DNA and Z-RNA (collectively known as ZNA) directly activate host responses. ZNA and the effectors it activates are both induced by infection and interferon [19,20,21]. ZNAs also arise from other sources of transcriptional dysregulation. The ZNA-forming sequences are embedded in EREs that lie just outside of gene boundaries or in introns [22,23,24,25,26,27,28,29].

There are two challenges for any system that depends on sensing ERE expression to initiate host defenses. Firstly, in normal cells, the system must be checked to ensure that any ERE sequences in normal transcripts do not activate immune responses. Secondly, the response must be escalatable when cells are under attack. Surprisingly, only two proteins are known to modulate this defense in humans. Both proteins possess a Zα domain that binds to ZNA with nanomolar affinity [30,31]. The first protein is p150, an interferon-induced isoform of the double-stranded RNA (dsRNA) editing enzyme ADAR, which deaminates adenosine to produce inosine [32]. The second protein, Z-DNA binding protein 1 (ZBP1) has two Zα domains. Its expression can increase by over 100-fold in the presence of interferon, leading to activation of the RIPK3-induced inflammatory cell death pathway, known as necroptosis [19,20]. The pathway eliminates infected or dysfunctional cells. Docking of ADAR p150 to Z-RNA formed in dsRNAs derived from EREs squelches the activation of ZBP1 [25,33,34,35]. Genetic evidence reveals that the ADAR Zα domain also negatively regulates the type I interferon response induced by the dsRNA sensor MDA5 (encoded by IFIH1) [36]. Editing of dsRNA by ADAR renders the mRNA susceptible to inosine-specific nucleases, leading to its removal [37]. The regulatory system using the Zα domains of ADAR and ZBP1 dates back to the earliest known eukaryotic progenitor [26].

#### 1.1.3. Alternative Processing of Krüppel Domain Protein RNAs

Protein-based defenses against retroelements have also evolved. In these proteins, a zinc-finger DNA-binding motif is paired with a Krüppel domain (KZBP) that represses gene expression. Over time, the family of zinc-family proteins has evolved to target EREs. There are six major clusters of KZFP on chromosome 19 that serve as hotspots for the generation of novel ZFPs, either through sequence variation, alternative splicing, or RNA editing [38,39,40]. The recombinants and variants generated are then subject to intense selective pressure. The form of selection is likely rapid, more rapid than the previous estimate of 7 million years [41]. Genomes unable to suppress TE expansion are less likely to survive in the population [39].

#### 1.1.4. AGO Proteins and Nuclear miRNAs

Another defense against EREs uses microRNAs encoded by host genes or within introns. The miRNAs target AGO proteins to ERE transcripts, leading to their rapid degradation or translational suppression. The mechanism is exemplified by the repeat-associated small RNAs, which are induced by retinoic acid and target AGO3 to EREs [42]. The different pathways involved are now well studied at the molecular level. Several clinically used therapeutic agents work through the RNA interference (RNAi) pathway. These small RNAs act in the cytoplasm.

Questions still remain about the nuclear roles of AGOs in tetrapods. Nuclear RNA interference in human cells has been demonstrated, resulting in lowered transcript levels and promoter methylation [43]. The nuclear localization of AGO2 is higher in confluent human cells, under stress, and during cellular senescence [44,45,46]. In particular, nuclear AGO2 is associated with suppression of EREs in resting mouse splenocytes [47]. Furthermore, the nuclear localization of AGO1X, an AGO1 isoform in which a C-terminal extension results from readthrough of the canonical stop codon, is associated with increased dsRNA production and induction of interferon [48]. The presence of all human AGO family members in both nucleoplasm and chromatin fractions, along with the accessory proteins required for RNAi, has been demonstrated by careful fractionation studies [49,50,51]. Proximity ligation of nuclear miRNA to mRNAs shows enrichment of interactions on the 3′ UTR, similar to the localization in cytoplasmic RNAs [51,52]. Other nuclear miRNAs are reported to alter transcript splicing [50].

### 1.2. EREs as Functional Elements in the Genome

Intriguingly, miRNA- and piRNA-mediated defenses are thought to derive originally from ERE sequences, enabling their suppression through various AGO and PIWI effector pathways [53,54]. However, metazoan EREs have also evolved at the same time. Some have been co-opted to control the programming of cellular responses. Their sequences are enriched in enhancers and promoters. EREs, like solo long terminal repeats (LTRs) and short interspersed nuclear elements (SINEs), have abstracted flipon sequences that modulate gene expression and RNA processing. The outcome is based on both their sequence and the structures they form. Through retrotransposition, they have spread transcription factor (TF) binding sites and flipon sequences that they adventitiously acquired throughout the genome.

#### 1.2.1. EREs and eRNAs

ERE-derived transcripts now play a role in gene regulation. They comprise over 30% of the long non-coding RNAs (lncRNAs) expressed in humans [15]. These sequences are enriched for long terminal repeat elements [55]. The ERE sequences are also highly enriched in enhancer RNAs (eRNAs) and play key roles in modulating gene expression. In experimental studies using proximity ligation assays, 37.9% of interactions between enhancer and promoter regions are associated with the hybridization of ALU elements [56]. In another example, G-quadruplex-forming promoter and enhancer transcripts are likely derived from EREs [57,58]. Additionally, eRNAs are thought to form TRX by interacting with promoter DNA duplexes. The TRX can turn transcription on or off, as reported for the dihydrofolate reductase (*DHFR*) gene [59], sphingosine kinase 1 (*SPHK1*) gene [60], the human β-globin locus [61], the Breast Cancer Susceptibility Gene 1 (*BRCA1*) promoter [62], and many other genes [63].

#### 1.2.2. EREs and Triplex Formation

The exact mechanisms involved in TRX-mediated modulation of gene expression vary. The effect differs with the junction formed between a TRX and B-DNA. The binding of a polypyrimidine third strand to a duplex with a polypurine-rich sense strand favors RNA polymerase engagement by creating a partially single-stranded junction. Transcription is reduced when the polypurine sequence is instead placed on the template strand [64]. In other cases, TRXs may enhance transcription by localizing histone acetylation complexes, as in the case of *SPHK1* [60], or diminish gene expression by competing away transcription factors, as reported for the trans-interaction of CISA with the *BRCA1* promoter [62]. In another example, TRX formation by pRNA at murine ribosomal RNA promoters leads to DNA methylation and reduced transcription [65,66]. Similarly, TRX formation downregulates transcription of the *DHFR* gene [59]. In this case, the lncRNA produced from the promoter (P0) located upstream of the primary promoter (P1) forms a TRX that silences the gene by interfering with the docking of transcription factor 2B to P1. In other cases, the lncRNA is first processed into miRNAs that act at more distant promoters, including those on different chromosomes, as shown for the lncRNA H19, which encodes miR-675 [67,68,69,70].

Some lncRNAs can act both in cis and trans. This outcome is true for the chromatin-associated antisense non-coding RNA in the *INK4* locus (ANRIL) lncRNA that recruits the polycomb repressive complex 1 (PRC1) to the *INK4a*/*ARF* locus [71]. ANRIL interacts in trans with 3227 loci in HeLa cells, with 65 genes activated and 123 silenced [72]. ANRIL also regulates in cis the locus from which it is transcribed [73]. The trans interactions of ANRIL map to a 21-base pair sequence with a G/A motif capable of TRX formation [74].

### 1.3. ERE Transcripts, Triplexes, and Chromatin

Central to these outcomes is the chromosomal architecture crafted with noncoding RNAs. Here, ERE transcripts play a central role. The extent of their involvement is revealed by studies that use fluorescent probes for in situ hybridization (FISH) to examine the distribution of LINE and SINE DNA sequences in the nucleus. The staining patterns reveal that long interspersed nuclear elements (LINEs) and SINEs are enriched in different nuclear compartments. Compartment A consists of active euchromatin and is enriched in SINEs. In contrast, LINES accumulate in compartment B, where heterochromatin is found, close to the nucleolar and nuclear membranes [75]. These compartments coalesce following RNase digestion, suggesting that RNA transcripts scaffold their separation [76]. Careful fractionation studies further confirm this finding. It is found that ERE transcripts are tightly associated with the insoluble chromatin matrix responsible for nuclear compartmentalization [77]. Of the chromatin-associated RNAs (caRNAs), about one-third contained repeat sequences, with one-third of these mapping to LINEs and another third to SINEs. Over 80% of the caRNAs map to introns and over 70% to protein-coding genes. Further, elongated transcripts that incorporate sequences downstream of genes (DOG) into mRNA, but lack poly(A) tails, are associated with chromatin. Their transcription is upregulated by osmotic stress, potentially rendering the nuclear scaffold resistant to rupture [78]. Similarly, in *Drosophila melanogaster*, over 95% of the core matrix attachment points within heterochromatin are associated with repeats [79], supporting a key role for these elements in forming the nuclear scaffold.

## 2. Results

### 2.1. Nuclear Scaffolds

To investigate how caRNAs influence nuclear structures, nuclear matrix-associated proteins were identified. The binding of one of these proteins, SAF-A (scaffold attachment factor-A, later identified as HNRNPU (heterogeneous ribonucleoprotein-U), was found to be dependent on transcription [80]. The caRNAs to which HNRNPU binds accumulate in non-dividing cells over time, thereby enabling the protein to remain stably attached to the matrix [81]. Over 90% of HNRNPU-bound sites are in compartment A. They are enriched in regions where chromatin loops are tethered to the matrix. Often, these sites are associated with the CCTC-binding protein (CTCF) [82]. The interaction between HNRNPU and the matrix is disrupted by cell division or RNase treatment. Potentially, cell division allows a reset of cell state, with the outcome reflecting where the matrix first nucleates.

**Figure 1:**
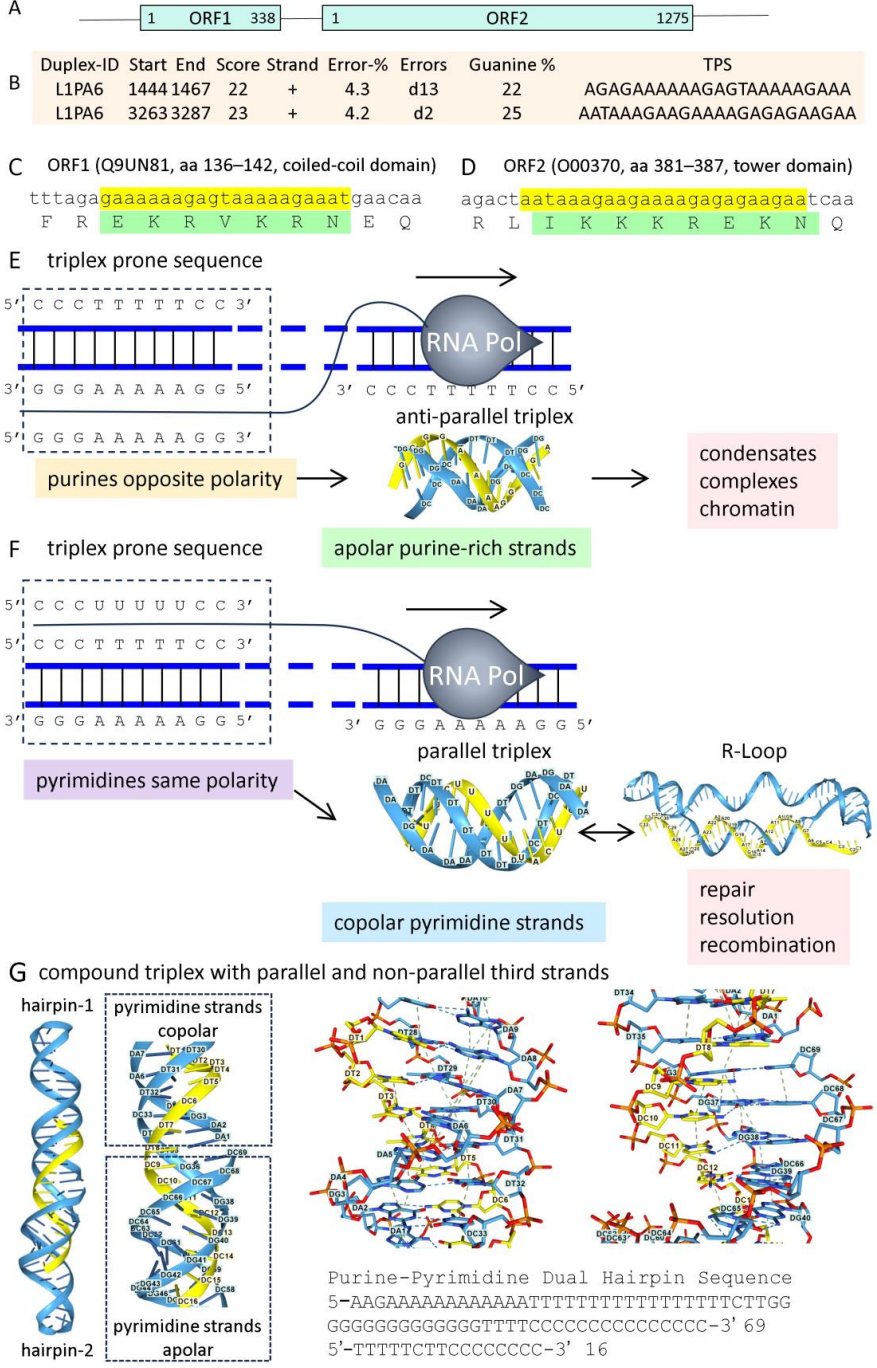
Triplex-forming sequences and folds. Retrotransposons contain conserved sequences within open reading frames (ORF) 1 and 2. (**A**) The positions of *ORF1* and *ORF2* in the L1PA6 long terminal repeat endogenous retroelement. (**B**) Location of the potential triplex-prone sequences (TTS) on L1PA6. (**C**,**D**) The mapping of the TTS in *ORF1* and *ORF2* to the amino acid sequence. (**E**) Formation of a triplex with an RNA third strand generates a structure where the purine strands are anti-parallel with the opposite polarity (apolar). (**F**) It is also possible for the RNA third strand to align where the pyrimidine transcript is parallel to the purine strand and copolar with the same polarity as the pyrimidine strand. If the RNA strand invaded the duplex, a DNA:RNA hybrid (DRH) displaces the other DNA strand to form a R-loop. In the text, these alternative outcomes are collectively referred to as RDLs, since it is sometimes a puzzle as to which is formed. (**G**) Compound triplexes that have both copolar (upper dotted box) and apolar (lower dotted box) third strands are possible. In the triplex structures shown, DNA strands are colored blue and RNA strands yellow. Processes associated with the different triplex folds are highlighted in the colored boxes (**E**,**F**).

The binding of HNRNPU to both RNA and DNA [80], combined with the lack of sequence specificity and the absence of a canonical RNA-binding domain, raised the possibility that this protein binds to a nucleic acid structure rather than a sequence motif. Indeed, the C-terminal domain is RGG-rich and has been reported to interact with G-quadruplexes [83,84,85,86]. The interactions with GQ may explain the 80% overlap in binding sites for HNRNPU, CCCTC-binding factor (CTCF), and the double-strand-break repair protein homolog (RAD21), which anchor DNA loops in mouse hepatocytes [82]. CTCF also docks to GQ DNA [87]. However, depletion of HNRNPU does not disrupt caRNA, suggesting that rather than tethering RNA to the matrix, HNRNPU binds to another preformed structure [88]. The presence of TRX-prone sequences, such as the AT-rich patches typical of matrix attachment regions (MARs) [77,89], suggested that the structure was a triplex. This possibility was supported by the enhancement of MAR formation by negative supercoiling [90]. Furthermore, computational analysis using the Triplexator and Triplex Domain finder algorithms implemented in the PATO utility reveals that the MARs conserved EREs incorporate triplex-forming sequences (Supplemental Data S1) [91,92]. An example of triplex formation by the LINE LPR6 element is presented in Figure 1A. These sequences map to open reading frames (ORFs) 1 and 2 and are within highly conserved protein-coding sequences.

ERE sequences are also found in many lncRNAs that are enriched in TRX-forming sites. Of the 49,372 high-confidence, noncoding genes collated at Lncipedia [93], 5842 contain a TRX-prone sequence of 30 bases or longer, and 24,675 (49.98%) have a sequence equal to or exceeding 15 bases (Supplemental Data File S2). The longest TRX is 627 bases and is found in lnc-PFKFB3-12:2. The larger TRXs potentially contribute to nuclear structure. The shorter segments in other lncRNAs are likely more amenable to modulating gene expression in cis or trans.

### 2.2. A Note on Copolar and Apolar TRX

By convention, the naming of TRX describes the relationship of the third strand to the purine-rich strand: in pyrimidine triplexes (R:Y*Y), the polarity of the third strand is parallel to the purine strand, whereas in purine triplexes (R:Y*R) it is anti-parallel (Figure 1E,F, “:“ refers to Watson-Crick hydrogen bonds, “*” to Hoogsteen bonds [9]). In these cases, the two copies of the purine and pyrimidine strands are oriented in opposite directions (referred to here as anti-polar or apolar). However, this scheme fails to capture the full complexity of TRX formation: stable triplexes can form from Y:R*R where the purine strands are parallel to each other (referred to here as copolar), not anti-parallel as assumed by the standard nomenclature [94]. Likewise, the Y:R*Y TRX with copolar pyrimidine strands has also been described [95]. In addition, “mixed sequences” of guanine and thymine can bind in either a parallel or anti-parallel mode, with the third strand copolar or apolar, depending on their composition [96]. In each of these docking modes, hydrogen bonding of the third strand can be optimized in two ways, producing either “directional” or “conformational” triplex isomers. “Directional” isomers have Hoogsteen base-pairing in parallel TRX and reverse-Hoogsteen in anti-parallel TRX. “Conformational” isomers differ in the rotation of the glycosidic bond, with the bases adopting the anti-conformation, pointing away from the sugar, or the syn-conformation, placing them over the sugar. The pyrimidine syn conformation is disfavored due to a steric clash of the C2 exocyclic group with the 2′-hydroxyl group of the sugar. Furthermore, the various possible TRX interactions can involve non-canonical hydrogen-bonding schemes. In some cases, TRX stability depends more on strong base-stacking interactions than on the number of hydrogen bonds formed [94].

Experimental studies of parallel-strand duplex DNA provide insight into the bonding schemes necessary to form copolar TRX. These duplex structures are stabilized by Hoogsteen base pairing rather than by reverse Watson–Crick bonding [97]. Their formation in vivo has been reported in *Drosophila melanogaster* and *Escherichia coli* [98,99,100]. It is also possible to synthesize oligonucleotides that form parallel-strand DNA duplexes and copolar rY:dR*rY triplexes that are more stable than their B-DNA and apolar counterparts [101,102]. Magnetic tweezer experiments also reveal that the stretching of a single-stranded DNA (ssDNA) enhances TRX formation with a plasmid containing a homologous sequence. Under these conditions, the duplex is not open, creating an energetic barrier that impedes ssDNA invasion. The TRX formed is copolar and accommodates many different sequences. The pairing occurs in the absence of protein and is likely an early step in recombination and repair reactions mediated by RECA family members [103].

### 2.3. RNA:DNA Triplexes

RNA:DNA duplexes differ in their in vitro ability to form triplexes, with rA:dT duplexes more stable than those composed of dA:dT base pairs, while rU:dA is the least stable of all [94,104,105]. Triplexes can also tolerate sequence mismatches by forming non-canonical base-pairing, such as C:G*T and G:C*A triplets. This underlying complexity has also been noted in genome-wide studies of triplex-forming sequences. Around 50% of TRXs were of mixed composition, in which the RNA sequences are capable of binding a particular DNA duplex in either the parallel or anti-parallel orientation. In particular, over 60% of triplex-forming RNAs isolated from the nucleus were from repeat elements, with the overlap rising to 80% in caRNAs. The triplex RNAs were enriched in super-enhancers and in anti-sense lncRNA transcripts [106].

Interestingly, compound TRXs can also be generated using AF3, which aligns parallel and anti-parallel folds with copolar and apolar third strands. This outcome is possible with colinear arrays of simple-sequence inverted repeats (Figure 1G). In other cases, three-stranded structures with a copolar alignment of an RNA transcript and its DNA counterpart are interpreted as evidence of an R-loop, in which an DNA:RNA hybrid (DRH) displaces the other DNA strand, not for TRX formation. Experimentally, the distinction is made by digestion with RNase H, which resolves the R-loops but does not digest a TRX. This result assumes that extended triple-stranded RNA/DNA complexes have not refolded during extraction from the cell. It also assumes that three-stranded structures are either an R-loop or a TRX, not a composite of both. The current techniques of isolation enrich for GC sequences, reflecting the bias of the reagents [107,108], or the extraction procedure with AT-rich sequences forming less stable DRHs, or instead folding as triplexes. Outcomes could vary with the RNA sequence, its length, and whether it is stretched [103]. With an extended length, TRXs with mismatches and sequence bubbles may be tolerated. The defects are offset by the numerous triplets formed. While the contribution of each triplet to the stability of the complex is small, they cumulatively act like a zipper to strengthen the TRX. The complexes themselves are dynamic and cycle between the different flipon states. Cellular proteins both stabilize and resolve each of the various structures to maintain the cycle. The composite structures composed of both R-loops and copular TRXs are referred to here as RDLs (RNA:DNA Lassos). The RDL will vary with length and sequence. Whether RDLs incorporate loops, triplexes, or both can be exploited by cells to trigger context-specific outcomes through what they capture.

### 2.4. Proteins That Bind Triplexes

Not much is known about TRX binding proteins. Although the biochemical assays described above suggest that TRX-binding proteins exist, there is a dearth of structural information on their interactions. There are many questions about how well in vitro studies of TRX-forming sequences reflect those that are active inside cells, as only a limited number of such studies have been published, all in the absence of proven TRX-binding proteins. Other screening methods based on chemical proteomics and triplex-forming probes have identified candidate TRX-binding proteins, for which a full detailed characterization has not yet been completed. Given the critical role of noncoding RNAs in cell biology, it would be helpful to understand how many of their interactions are mediated by TRX formation.

**Figure 2:**
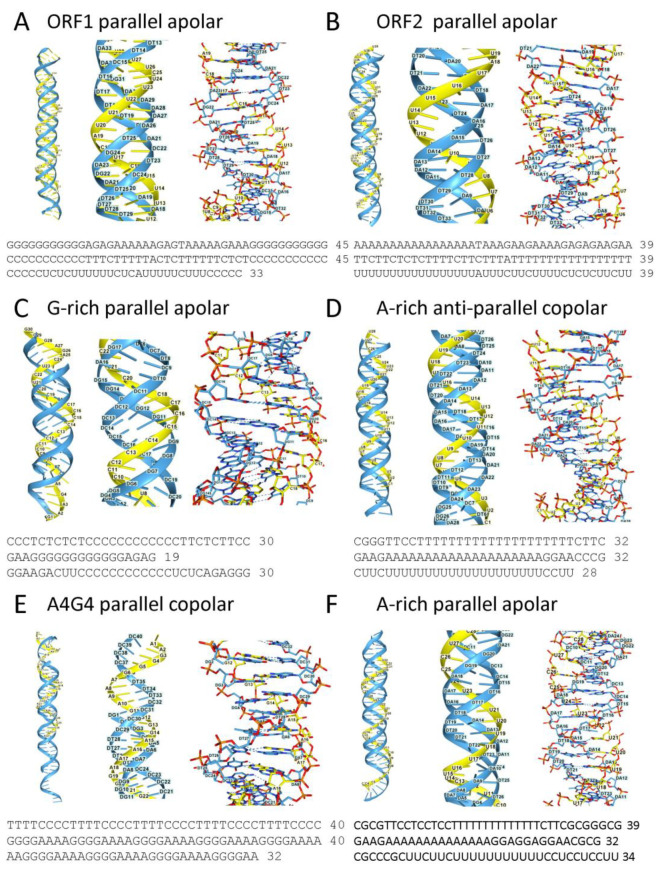
Examples of the triplexes folded in AF3 from a DNA duplex and an RNA third strand. These models are also used in the subsequent figures. (**A**,**B**) The folds of ORF1 and ORF2 TRXs from L1PA6 shown in Figure 1A–C. (**C**–**F**) Triplexes with different sequences and lengths can fold as parallel, anti-parallel, apolar, and copolar. The sequences are given 5′ to 3′ with the two DNA strands above the RNA strand. In the triplex structures shown, DNA strands are colored blue and RNA strands yellow. The triplexes are shown as cartoons and as ball-and-stick models with standard coloring, with the hydrogen-bonding schemes depicted by dotted lines. DNA strands are colored blue and RNA strands yellow.

To address these challenges, models of the interaction between potential TRX-binding proteins and TRX in AF3 are presented. The outputs were visualized using NGL Viewer [109]. The first two TRX probes are derived from the L1PA6 retroelement sequences given in Figure 1A. Other TRX used in these models are shown in Figure 2. They are much longer than those usually studied in vitro. The TRXs modeled have different topologies. These include TRX from the LINE LPR6 *ORF1* and *ORF2* (Figure 2A,B), a G-rich parallel, apolar, and an A-rich parallel, copolar TRX (Figure 2C,D), the simple A4G4 repeat anti-parallel copolar TRX, and the mixed sequence, A-rich apolar TRX (Figure 2E,F). The analysis of TRX interactions with protein starts with structural proteins, then moves on to those that regulate gene expression, and includes proteins that modulate triplex formation. Models for HNRNPU, TP53, AGO, PIWI, and REL Domain proteins are presented, followed by those for chromatin remodelers, helicases, and topoisomerase. Models for core polycomb repressive complexes (PRC) 1 and 2 are then described. A summary of these interactions is presented in the final figure.

#### 2.4.1. HNRNPU Triplex Models

**Figure 3:**
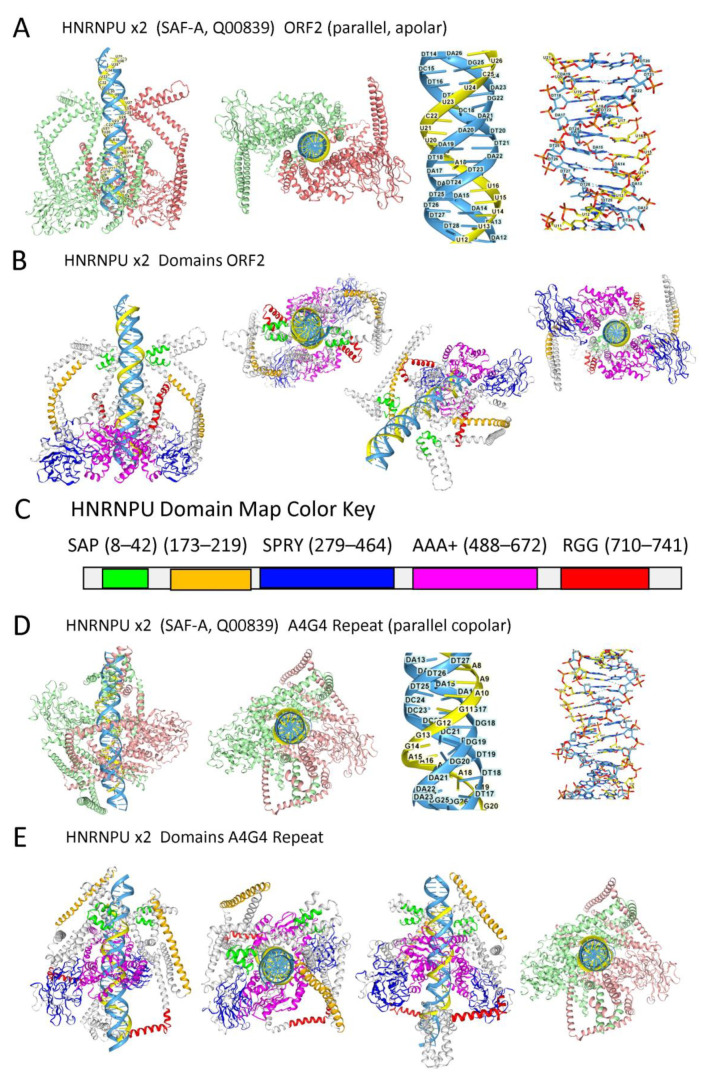
The interaction of two SAF-A proteins (colored green and brown and encoded by the *HNRNPU*) with the ORF2 TRX (**A**,**B**) and A4G4 TRXs (**D**,**E**) from Figure 1. Panels (**B**,**D**) color each SAF-A domain by the key given in (**C**). The bases in the TRX cartoons are numbered by strand. DNA strands are colored blue and RNA strands yellow. DNA bases are further denoted by “d”.

The binding of HNRNPU to a TRX was suggested by the presence of AT-rich patches typical of matrix attachment regions (MARs) [77,89], and the dependence of complex formation on RNA. AF3 reveals that HNRNPU can dock to both anti-parallel (Figure 3A,B) and parallel TRX (Figure 3D,E), with the HNRNPU domain boundaries illustrated in Figure 3C. Both TRX types have an RNA polypyrimidine third strand. HNRNPU is fully folded despite containing numerous IDRs. The TRXs differ in the conformation of the purine strand: syn when the pyrimidine strands are apolar and anti when they are copolar. In both triplexes, bifurcated hydrogen bonds are present. Mapping of the domains involved in the interaction of HNRNPU with the triplexes (Figure 3C) reveals that they are independent of the C-terminal RGG domain, which likely binds GQ [110]. The fold depends on the AAA+ domain, which contains Walker type A and B boxes. Previous studies have shown that this domain contributes to the ATP-dependent oligomerization of HNRNPU, which is thought to be necessary for chromatin net formation [80]. The findings support a model where both TRX and GQ flipons contribute to the nuclear matrix.

The exact role HNRNPU plays in the nucleus may vary depending on whether it binds to a copolar or an apolar TX. Both TRX types can form with lncRNAs. An apolar TRX requires the RNA to form a loop that reverses its orientation. In contrast, a copolar TRX could directly incorporate a gene transcript (Figure 1F). It has been widely assumed that in copolar situations, an RDL would displace the other DNA strand, forming an R-loop rather than a TRX. The ssDNA in an R-loop then becomes vulnerable to damage, increasing the probability of chromosomal breakage. Instead, a TRX stabilized by proteins such as HNRNPU or a three-stranded structure masked by other proteins would diminish those risks. TRX formation may confer additional benefits. The TRX would also pause a transcriptionally active RNA polymerase. No further RNA synthesis would occur until the obstruction was resolved, either by its removal, by the assembly of a pre-RNA processing complex, or by the formation of a matrix attachment site. EREs that promote copolar TRX formation would coordinate host responses as the context changes.

Apolar TRX could arise by folding back a mirror-image repeat (MIR) transcript on itself. MIR repeats comprise 1–2% of the genome [111] (Figure 1). Alternatively, an apolar TRX could form through trans-interactions with lncRNAs. The TRXs then serve to anchor other sequence motifs in the lncRNAs that set local chromatin state by scaffolding the assembly of protein complexes, rather than just suppressing ERE transcription.

#### 2.4.2. TP53 Triplex Models

The TP53 protein is another protein that has been recently found to suppress LINE1 loci through the abrogation of R-loops [112,113,114]. Studies in both *Drosophila melanogaster* and zebrafish show that this defense mechanism is ancient and works in conjunction with PIWI proteins. In mouse and human cancers, TP53 loss-of-function (LOF) mutations enhance retrotransposition [115]. TP53 is also reported to bind triplexes [116].

**Figure 4:**
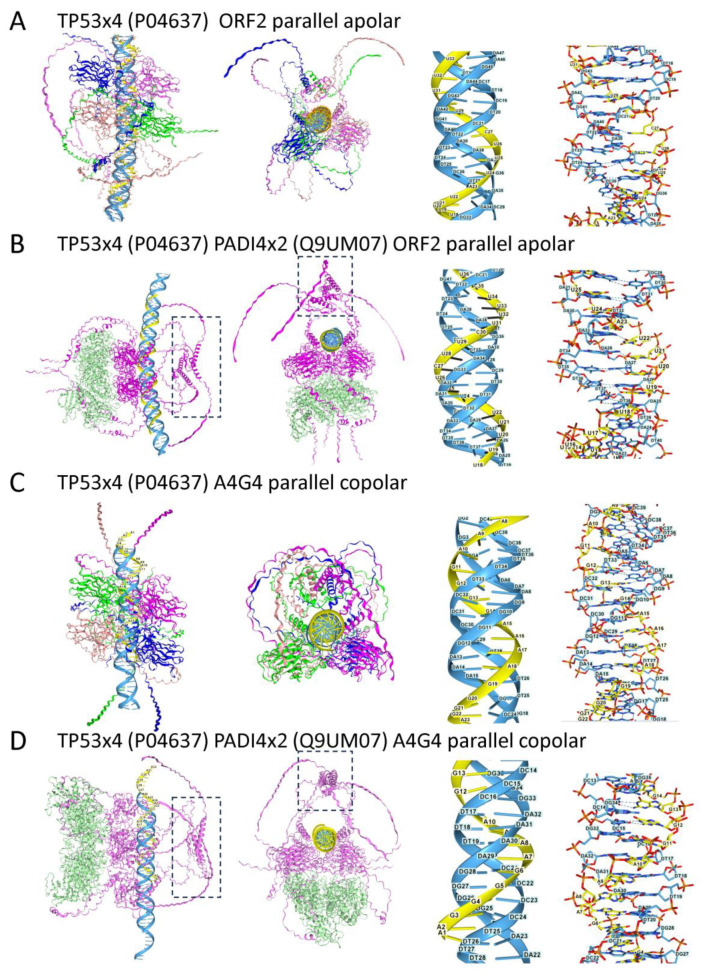
The interaction of the cellular tumor antigen p53 (TP53) tetramer with the ORF2 TRX (**A**,**B**) and with a d(A4G4) repeat. The four p53 proteins are colored differently. Triplexes and bonding interactions are drawn as in Figure 2. Docking of two protein-arginine deiminase type-4 (PADI4) to the tetramer is shown in (**C**,**D**). The p53 proteins are colored lilac, and the PADI4 proteins green. In Panel (**D**), the C-terminal tetramerization domain is within the dotted box. The TRX are shown as cartoons, with bases numbered by strand. DNA strands are colored blue and RNA strands yellow. DNA bases are further denoted by “d”. TRXs are also drawn as ball and stick representations using standard colors, with hydrogen bonds indicated by dotted lines.

AF3 models revealed that tetramers of TP53 protein (and the TP63 and TP73 family members also dock to both copolar and apolar TRX (Figure 4). The interaction maps to the DNA-binding domain, rather than to the C-terminal tetramerization domain, as previously reported [116]. The bound tetramers provide a landing site for peptidyl-arginine deiminase 4 (PADI4), thereby stabilizing the interaction. PADI4 is known to modify TP53 by converting lysines to citrulline. The citrullination of the p53 DNA binding domain is reported to inhibit TP53-mediated gene activation. In contrast, citrullination of the carboxy terminus activates and redirects TP53 to a subset of tumor suppressor genes that have promoters with E26 transformation-specific (ETS) transcription factor binding motifs [117,118,119]. In the complex shown in Figure 4, PADI4 is on the opposite side of the TRX to the C-terminal tetramerization domains that lie within the dashed boxes. The pose suggests that PADI4 stabilizes the TP53/TRX complex rather than modifying these TP53 residues. The potential ability of TRX to fix TP53 in place adds a different twist to the debate of whether TP53 is “smart” or “dumb” in its selection of binding sites [120]. Regardless of the mechanism, the docking of TP53 to TRX formed by nascent RNAs would block RNA polymerase engagement and impede transcript elongation. This activation-induced gene repression would also downregulate EREs, limiting the DNA damage they cause by transposing to other sites. Interestingly, the PADI4/TP53 c-terminal region engages SET domain containing 2 protein (SETD2) in AF3 models. SETD2 regulates both transcription and repair by adding three methyl groups to histone H3 at lysine 36 (H3K36me3) [121].

#### 2.4.3. AGO Triplex Models

Unlike lncRNAs, short RNAs, such as piRNAs and miRNAs, act only in trans [115]. It has been proposed that some miRNAs guide AGO proteins to their targets by TRX formation [122,123,124]. Further, it was suggested that such TRX are stabilized by histone H3, but not histone H4, tails [125]. The TRX may help localize nucleosomes, creating open DNA regions that enable transcription factor binding and promote the cycling of flipon conformations. It has also been reported that AGO-mediated RNAi can be induced by copolar, parallel-strand DRHs [126]. No structural information currently exists on the nature of the AGO interactions with parallel strand DRHs or TRXs.

Although the cytoplasmic roles of miRNAs in negatively regulating mRNAs are well established, the functions of nuclear miRNAs remain an area of active research. In plants, miRNAs induce DNA methylation [127], while in *Schizosaccharomyces pombe*, they promote heterochromatin formation [128]. In other cases, miRNAs produce gene activation. This outcome was first suggested by the use of antagomirs that inhibited miR-122 activity. As hypothesized, administration of an antagomir miR-122 inhibitor to mice increased expression of genes enriched for miR-122 target sites in their 3′ UTR [129]. Surprisingly, other genes lacking miR-122 target sites in their 3′ UTRs showed diminished expression. Later studies revealed that the direct interaction of AGO1 or AGO2 miRNA mimetics could increase the expression of *CHD1* (E-cadherin), *VEGF*, *CDKN1A* (p21), and *CEBPA* genes [130,131,132].

A variety of mechanisms have been hypothesized to explain this result. The proposed interactions include targeting an antisense RNA to inhibit transcription; binding to the sense transcript to initiate a transcription complex; and formation of an R-loop or a TRX. Others have suggested that AGO acts indirectly by modulating protein complex formation, rather than by directly contacting DNA. Those models were based on the AGO1 p.Y527E variant, which is unable to bind miRNAs, but can still rescue estrogen-dependent transcription in MCF7 cells upon depletion of endogenous AGO1 [133]. In contrast, localization of AGO1 to enhancers is reported to depend on active transcription and the production of enhancer RNAs (eRNA) [49]. Furthermore, depletion of AGO1 disrupted the boundaries between the active Compartment A and the inactive Compartment B, indicating that this protein can help set the chromatin structure [134]. In such a scenario, AGO1 can reestablish compartments after their loss during cell division, acting before the nuclear membrane reforms and thereby enabling an update based on the current cellular context.

**Figure 5:**
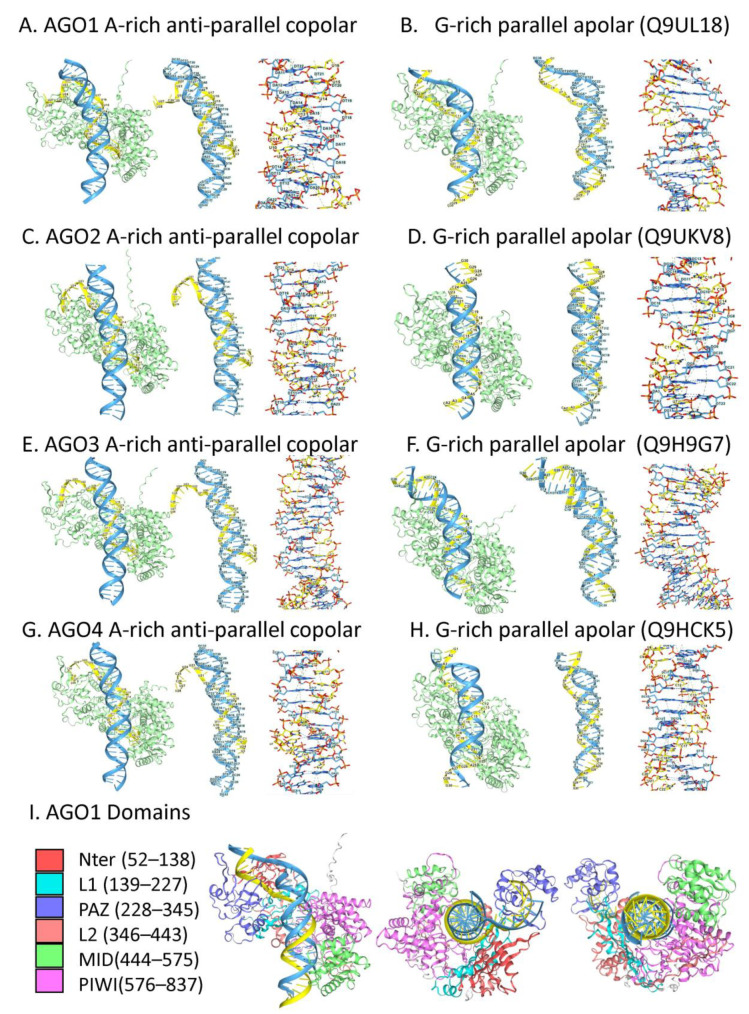
The docking of AGO 1–4 proteins to either an A-rich anti-parallel copolar triplex (left panels) or a G-rich parallel apolar triplex is shown in panels (**A**–**H**). Triplexes and bonding interactions are drawn as in Figure 2. In Panel (**I**), the AGO1 domains are colored to highlight those involved in docking to the triplex in Panel (**B**). The TRX are shown as cartoons, with bases numbered by strand. DNA strands are colored blue and RNA strands yellow. DNA bases are further denoted by “d”. TRXs are also drawn as ball and stick representations using standard colors, with hydrogen bonds indicated by dotted lines.

The potential for AGO protein docking to copolar and apolar TRX was modeled in AF3. The models used RNAs as the third strand (Figure 2C,D). Three questions were investigated. Do any or all human AGO proteins bind TRX as predicted from experimental data? Are the interactions affected by the AGO1 p.Y527E variant or equivalent variants in other AGO proteins? Can TRX form with non-canonical sequences that lack a classical purine/pyrimidine repeat motif? The models confirm that all four human AGO proteins dock to triplexes in both parallel and anti-parallel, copolar, and apolar conformations (Figure 5). The contacts between the AGO proteins and TRXs are extensive and involve both the PAZ and PIWI domains (Figure 5I). The formation of the complexes was not affected by the AGO1 p.Y527E variant, or by the equivalent variants in other AGO proteins. The finding suggests that the nuclear AGO proteins act through pathways different from those used in the cytoplasm. With an apolar TRX, formation of an R-loop is disfavored as strand invasion would result in a parallel-stranded duplex. Instead, TRX formation likely supports the assembly of a regulatory protein complex. In contrast, a copolar TRX facilitates strand invasion and DRH formation. Subsequent processing of the DRH by the DICER endonuclease, by PIWI proteins with slicer, or RNase H activity, can then yield small regulatory RNAs [17,135,136]. Copolar TRXs may also impact the formation of ANS by other flipons. As shown by AF3 models, both G- and Z-flipon motifs can be stabilized as TRXs by AGO and PIWI proteins, provided the d(G)n and alternating d(CG)n and d(TG)n repeats are short. The findings suggest that AGO proteins can modulate gene expression by favoring the formation of TRXs in promoters rather than GQ or Z-DNA structures [137].

While miRNA may be one source of guides, nascent RNAs and lncRNAs, or the products formed by fragmentation of prematurely-terminated transcripts may represent another way to modulate gene expression through TRXs formed with RNA as the third strand. The TRXs may help position nucleosomes to move promoters into or out of open chromatin regions, and to localize protein complexes that epigenetically set chromatin marks. The enrichment of AGO1 and AGO2 at promoters in the Encyclopedia of DNA Elements (ENCODE) datasets is consistent with these different outcomes [137].

#### 2.4.4. PIWI Triplex Models

**Figure 6:**
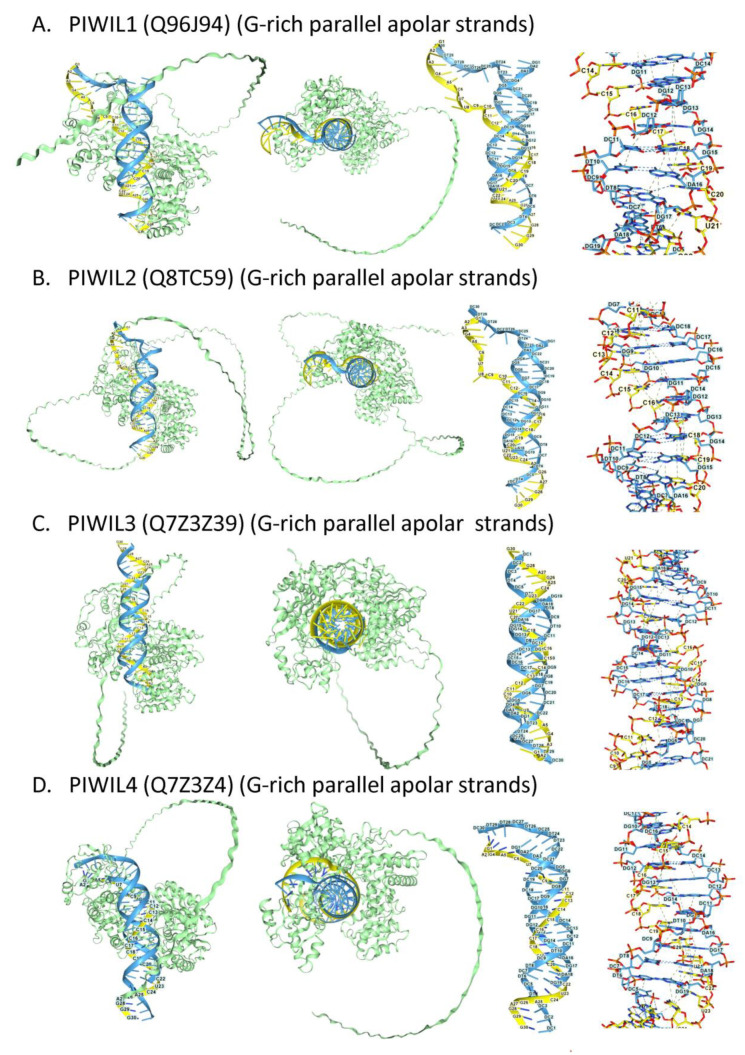
The docking of human PIWI-like proteins 1–4 to a G-rich parallel apolar triplex is shown in panels (**A**–**D**). The TRX are shown as cartoons, with bases numbered by strand. DNA strands are colored blue and RNA strands yellow. DNA bases are further denoted by “d”. TRXs are also drawn as ball and stick representations using standard colors, with hydrogen bonds indicated by dotted lines.

Human PIWI-Like (PIWIL) proteins were also found to dock to copolar and apolar TRX (Figure 6), supporting a role in targeting ERE sequences capable of TRX formation. Only the classical apolar TRX are shown [138]. Interestingly, PIWIL4 expression is enhanced in acute myeloid leukemia (AML). Besides piRNA-guided interactions, PIWIL4 is reported to suppress R-loop formation, maintain genome integrity, and promote disease progression [136]. These outcomes depend on a PIWIL4 RNase H catalytic activity. In such cases, resolution of a copolar TRX to form a DRH would generate an RNase H substrate. Around half of the PIWIL4-associated RNA in AML mapped to pre-mRNA and mRNA protein-coding genomic regions (48%), while 17% mapped to eRNAs. The piRNAs were enriched for an AGAGAGA TRX-prone motif. Amplification of piRNAs could then proceed by the PPPi pairing of sense-antisense transcripts produced from the same gene, trans interactions with lncRNA fragments, and small ncRNAs arising from a related pseudogene or paralog. In such scenarios, PIWIL4 will slice single-stranded RNA 10 bases upstream of where the 5′-phosphorylated piRNA binds. 

#### 2.4.5. REL Domain Triplexes

While both miRNAs and piRNAs can suppress EREs, a different mechanism for negatively regulating active LINEs involves REL domain proteins from the Nuclear Factor-Kappa-B (NF-KB) family. This outcome is exemplified by the p50 (encoded by the *NFKB1* gene), which produces epigenetic modifications in mouse hemopoietic stem cell genes that contain the intronic *L21Md LINE* [139]. This pathway acts both in quiescent cells and during the resolution of interferon responses to produce histone lysine 9 trimethylation (H3K9me3). The modification paradoxically leads to lower gene expression, as H3K9me3 impairs RNA polymerase elongation, but results in a more stable transcript due to suppression of alternative splicing. The pause in transcription is beneficial, as it allows more time for pre-mRNA maturation. Consequently, hypomethylated *LINEs* are more highly transcribed, with pre-mRNA splicing defective, leading to rapid triage of the transcript [140,141]. 

Both p50 (a proteolytic cleavage product of a p105 precursor) and p52 (processed from p100, and encoded by *NFKB2*) form homodimers and heterodimers with other family members, including RELA (p65), RELB, and c-REL, that possess the transactivation domains they lack. The different dimer combinations underlie a complex regulatory system. Interestingly, the sites of H3K9me3 in gene bodies are enriched for both RELA (p65) and heterochromatin protein 1 (HP1), which has protein isoforms encoded by the chromobox encoding genes CBX1, CBX3, CBX5, and also for DDX17 localization [142,143]. In mice, RELA-bound genes include *EiF2ak2*, *Oas1g*, *Cd274* (encodes PD-L1), and *Cd86*. In humans, binding sites for p65, p52, and RELB proteins are also enriched in ALUSx, ALUSg, and ALUY family members [144]. 

REL domain proteins also affect gene transcription by engaging both promoters and enhancers. At promoters, the competition between RELA and RELB regulates the expression of inflammatory pathways, thereby dampening autoimmune reactions [145]. The competition may also affect RELA-mediated suppression of intronic EREs and the eRNAs they encode via targeted H3K9me3 modification. When this process is dysregulated by viral infections, during tumorigenesis, and within senescent cells, transcription of ERE results in the formation of dsRNAs and Z-RNAs that protect the host against the threats posed by initiating cell death [27].

**Figure 7:**
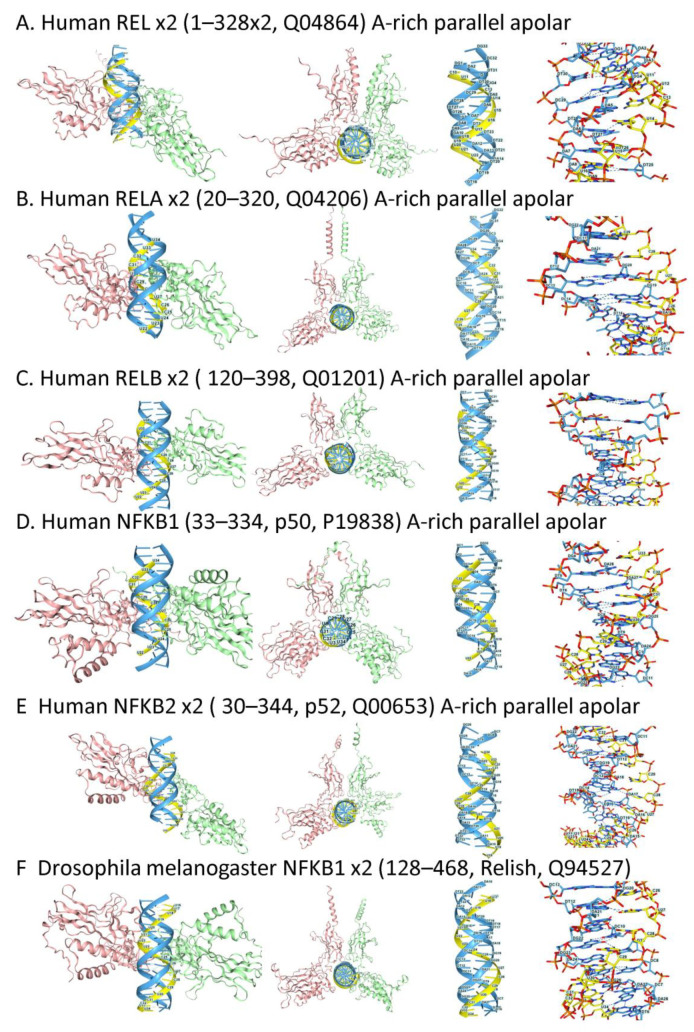
The docking of the nuclear factor-kappa-B family (NFKB) of REL domain proteins to an A-rich parallel apolar triplex is shown. (**A**–**E**). Human REL domain proteins. (**F**). The relish protein of *D*. *melanogaster*. The TRX are shown as cartoons, with bases numbered by strand. DNA strands are colored blue and RNA strands yellow. DNA bases are further denoted by “d”. TRXs are also drawn as ball and stick representations using standard colors, with hydrogen bonds indicated by dotted lines.

H3K9me3 sites within ERE are close to RELA and HP1 motifs, with HP1 sites enriched for TRX forming GAGAGA sequences. It was therefore of interest to test whether REL proteins docked to TRX. AF3 modeling revealed that all human REL domains docked as homodimers to the parallel, apolar A-rich TRX (Figure 7A–E). The *D*. *melanogaster* relish protein, an ortholog of vertebrate family members, also bound to a TRX (Figure 7F). The results raise the interesting possibility that TRX formation by ERE contributes to NF-KB regulatory circuits. Previously, R-loop formation induced by strand-invasive modified oligonucleotides has been used to inhibit NF-KB inflammatory gene transcription [146]. Notably, the sequence-specific oligos are not expected to disrupt the binding of REL proteins to TRX, nor the subsequent H3K9me3-mediated suppression of EREs. Only when active EREs produce more TRXs than REL proteins can bind will this mechanism fail [27].

### 2.5. Proteins That Modulate Triplexes

**Figure 8:**
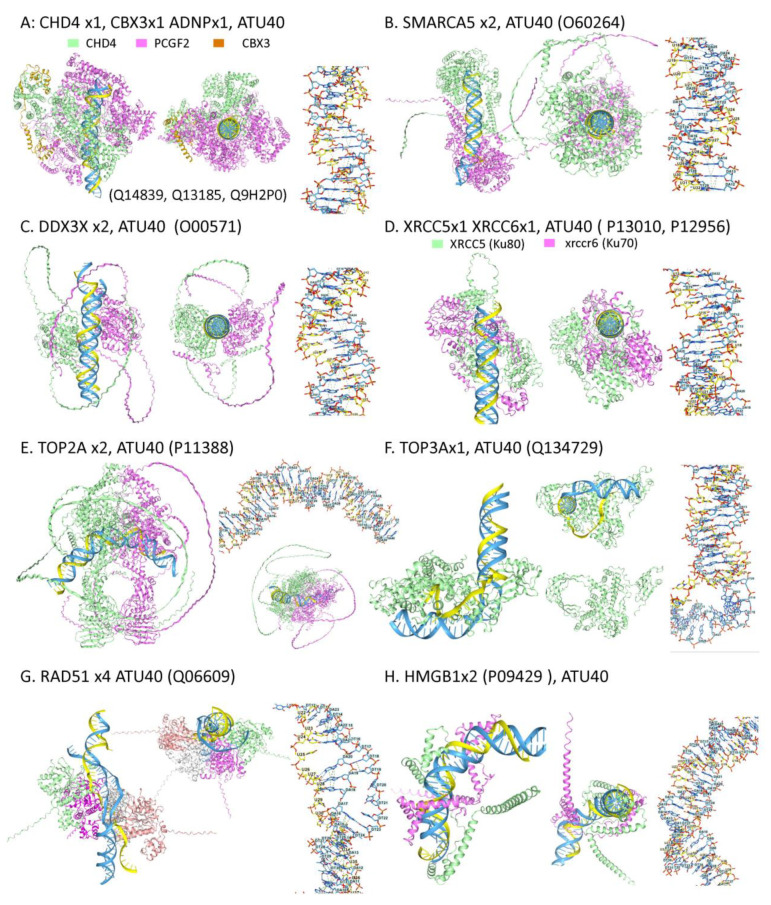
Triplex interactions with architectural proteins previously identified in proteomic screens. (**A**) CHD4 chromatin remodeler, helicases. (**B**) The SWI/SNF-related matrix-associated actin-dependent regulator of chromatin subfamily A member 5 (SMARCA5) is a component of the Imitation Switch chromatin remodeler. (**C**) DDX3X helicase. (**D**) KU70 and KU80 complex formed by X-ray repair cross-complementing proteins 5 and 6 (XRCC5, XRCC6) (**E**) A dimer of topoisomerases 2A, with each chain colored separately. (**F**) A topoisomerase 3A passes the duplex through a pore while peeling off the RNA. (**G**) A repair recombinase RAD51 tetramer bound to a three-stranded RDL. (**H**) Bending of a triplex by two bound high mobility group B1 proteins (HMGB1). The TRX are shown as cartoons, with bases numbered by strand. DNA strands are colored blue and RNA strands yellow. DNA bases are further denoted by “d”. TRXs are also drawn as ball and stick representations using standard colors, with hydrogen bonds indicated by dotted lines.

Like other flipons, the dynamic formation and resolution of TRXs can reset cellular programs. These outcomes depend on proteins that maintain the B-DNA duplex in a low-energy state. The pathways involve chromatin remodelers, maintenance and repair helicases, topoisomerases, and strand-exchange proteins (Figure 8). The targeting of many of these proteins to TRXs is supported by experimental evidence. A chemoproteomic screen revealed that CHD4, SMARCA5, XRCC5, DDX3X, TOP2a, and HNRNPU interact with TRXs [147]. Another screen confirmed interaction with XCCR5 and identified a further interaction with TOP3A [148]. TRX formation by LncRNAs can seed the assembly of repressive protein complexes, leading to heterochromatin formation. For example, the ANRIL lncRNA regulates the *INK4a/ARF* locus by localizing PRC1 through an interaction with CBX7 [71]. Other lncRNAs localize PRC2 through TRX formation [63]. The in vivo screens may have had a limited ability to capture these interactions due to the compact, inaccessible nature of heterochromatin, which restricts probe access.

It was therefore of interest to use AF3 models to assess whether these candidate proteins could dock to TRX (Figure 8). Given the enrichment of AT-rich sequences in both scaffold attachment and ERE sequences, a d(A:T)*rU triplex of 40 bases (ATU40) was used to test each prediction. Models are presented for the chromodomain (CHD4) and imitation switch (ISWI) chromatin remodeling (CRM) complexes, for topoisomerase 2a and 3A (TOP2A and TOP3A), and for the PRC1 and PRC2 complexes.

#### 2.5.1. CHD4 Chromatin Remodelers

CHD4 is an essential protein that is a component of nucleosome remodeling and the deacetylase (NuRD) complex that silences active promoters [149]. This complex deposits the replication-independent histone 2A variants H2A.Z.1 and H2A.Z.2 (encoded by *H2AFZ* and *H2AFV*, respectively) at its sites of action. CHD4 also forms the ChAHP complex with the activity-dependent neuroprotective protein (ADNP) and one of the HP1 gene variants [150]. Docking of ChAHP renders the surrounding chromatin inaccessible, but does not produce H3K9me3-modified nucleosomes [150]. In mice, the ChAHP complex binds to a bipartite motif that is also bound by the CTCF protein. The motif consists of M1 and M2 sequences separated by a 2–21 basepair spacer. Chromatin loop formation is restricted by competition with the ChAHP complex [151]. The M1 motif (CCCTCTTCT) was spread throughout the mouse genome by SINE B2 retrotransposition [152], along with a downstream purine-rich sequence (AAAAAAAAAAAAAAGAAAGAAAAA) [153]. Both sequences can potentially form a TRX. A related ChANP2 complex that replaces ADNP with ADNP2 inhibits transcription factor IIIB (TFIIIB) recruitment to SINES but does not affect TFIIIC binding, as they dock to different sites [154,155].

AF3 models show that the ChAHP complex docks to a triplex, with complete folding of all IDRs (Figure 8A). The results suggest that TRX recruitment of the ChAHP chromatin remodeling complex prevents chromatin loop formation by displacing CTCF. Indeed, the loss of ChAHP, caused by *ADN*P deletion, disrupts the chromatin architecture and is associated with the ectopic localization of the CTCF protein. In embryonic stem cells, the deletion interferes with neuronal lineage differentiation [150].

ChAHP and ChANP2 binding sites are enriched in other types of repeat sequences, but no sequence-specific motif has been identified. The docking is thought to involve the zinc-finger domains (ZFDs), which differ between ADNP and ADNP2 [154]. Interestingly, the ZFDs have the potential to bind GQ, and the locations of GQ structural motifs within EREs differ. The docking of the ZFDs to a specific set of GQs could then mediate the competition among ADNP, ADNP2, and CTCF [156,157,158]. With EREs, the TRX and G-flipons interactions differ from those between HNRNPU and CTCF, which are involved in anchoring the nuclear matrix. However, both schemata support a conjoined role for TRX and G-flipon in regulating the chromatin state.

#### 2.5.2. Imitation Switch Chromatin Remodelers

The SMARCA5 protein (SNF2H) also docks to TRX in AF3 models (Figure 8B). SNF2H is an ATPase component of the imitation switch (ISWI) chromatin-remodeling complex that facilitates DNA replication, DNA repair, and transcription. The machinery maintains open chromatin by increasing the spacing between nucleosomes [159]. The process involves the sliding of nucleosomes along DNA rather than their eviction. Loss of SNF2H decreases nucleosomal phasing, increases linker lengths, and diminishes the formation of topologically active domains (TADs) but does not affect the organization of chromatin into compartments A and B [160,161]. Interestingly, SNF2L positions nucleosomes adjacent to CTCF and other transcription factors [160]. The depletion of SNF2H also increases the number of CTCF sites bound by nucleosomes.

The interaction of SNF2H with other CRMs is also essential in phasing nucleosome arrays, thereby defining active promoter and enhancer regions. Phasing helps prevent transcriptional interference of a promoter by transcription initiated from upstream or antisense transcription start sites. Readthrough by the elongating RNA polymerase complex disrupts the complexes formed at the affected promoter [59,162]. The placement of nucleosomes helps isolate promoters from one another and enables the switch from one to another as the context changes [9].

In humans, AT-rich regions play an important role in transcription. Poly(dA) tracts of exactly length five are enriched 5′ to transcription start sites, while polyadenylation transcription termination sites are depleted of nucleosomes [163,164]. A likely role for TRX is to set the boundaries for these regions. The open DNA regions formed permit the docking of transcription factors and the modulation of transcription through the cycling of Z- and G-flipon structures [165]. The phasing of nucleosomes is also essential for enhancer function, as these regions often contain many EREs. Interference from bidirectional transcription at these EREs can disrupt embryogenesis, as observed when chromatin remodelers fail to properly place nucleosomes [166].

#### 2.5.3. Helicases and TRXs

Helicases help resolve triplexes and other folds, such as GQ and hairpins [167]. The different helicases are optimized for specific contexts. Both DDX3X and KU70/KU80 (encoded by *XRCC5* and *XCCR6*) were identified in the chemoproteomic screen as TRX-binders [147]. DDX3X undergoes nucleocytoplasmic shuffling and affects all aspects of RNA biology. DDX3X is encoded on the X chromosome and has a paralog, DDX3Y, on the autosomal region of the Y chromosome. Mutations have been linked to several diseases and sex-specific outcomes, in part reflecting leaky X-inactivation of the gene [168]. KU70/KU80 is essential for the operation of the classical nonhomologous end-joining (c-NHEJ) DNA repair pathway and also binds RNA hairpins [169]. The complex recognizes a variety of alternative structures that can fit besides TRX into the central cavity of the ring it forms [170]. Interestingly, the KU70/KU80 promotes the truncation of LINE1 elements during retrotransposition, suggesting a role for TRX formation in inhibiting reverse transcription [171]. In AF3 modes, both the DDX3X and KU70/KU80 helicases engage a parallel, copolar TRX.

#### 2.5.4. Topoisomerases and TRX

AF3 also demonstrates the docking of the topoisomerases TOP2A and TOP3A to parallel, copolar TRXs. TOP2A binds to a bent TRX, while TOP3A appears to peel off the RNA strand by passing the duplex through a pore (Figure 8C,D). These enzymes can resolve transcription-related tangles that result from the winding of a pre-mRNA around the DNA helix. TOP2A cleaves both strands of a DNA duplex to permit passage of the RNA. This process occurs at sites where DNA duplexes cross over [172]. TOP2A is localized by an interaction with the AT-rich interactive domain-containing protein 1A (ARID1A) component of the SWI/SNF chromatin remodeler. Despite its name, ARID1A binds DNA without sequence-specificity [173,174]. Although the direct binding of TOP2A to triplex structures has not been experimentally investigated, the TOP2A poison pyridostatin also induces R-loops [175]. In AF3 models, TOP2B also docks in a similar mode to TRX. Interestingly, TOP2B binding sites at TAD boundaries overlap those for CTCF and cohesin complexes [176]. Computational studies with the Triplexator program also reveal significant enrichment of potential TRX-forming sequences with CTCF sites [177]. In this context, TOP2A may prevent DNA methylation at these sites by binding to TRXs, thereby masking them and facilitating CTCF recruitment once TRXs are resolved [178,179]. In contrast to TOP2A, TOP3A cleaves a single DNA strand near the sites of hemicatenate formation. A role for type I topoisomerase in resolving R-loop RDLs has been experimentally demonstrated [180].

#### 2.5.5. The RAD51 Recombinase and TRX

It was also of interest to test how RAD51, a human ortholog of the bacterial recombinase A (RECA) enzyme, interacted with an RDL composed of an RNA third strand and a DNA duplex [181]. Three-stranded DNA structures form during RAD51 homology-dependent DNA repair and recombination [182,183]. A displacement D-loop (rather than a R-loop) arises when the invading copolar strand unpairs the matching strand of the DNA duplex. The base pairs present at the beginning and end of the exchange are energetically equivalent, with directionality of exchange determined by other factors [14]. With RNA as the invading strand, docking rather than exchange may result. The rU:dA base pair is less stable than dT:dA, thereby disfavoring exchange. Further, the base-stacking of an rU repeat can stabilize a TRX intermediate, creating an additional energetic barrier to strand invasion of a duplex [94]. In AF3 models, it is possible to capture RAD51 bound to an extended, partially paired three-stranded structure. In such cases, RAD51 may not create an RDL but instead cloaks the complex, thereby decreasing the risk of DNA damage by restricting ssDNA formation. Genomic incorporation of retrotransposons appears not to be affected by CRISPR knockout of RAD51 [184]. However, RAD51 is recruited to sites of damage by CTCF and damage-induced AGO2-bound RNAs and may protect against the genomic instability induced when they are actively transcribed [185,186,187].

#### 2.5.6. HMGB1 and TRX

The high-mobility group (HMG) proteins have long been associated with DNA bending and looping. By displacing histone H1 from the nucleosome, they enable the formation of open chromatin structures. The HMG proteins contain a DNA-binding box that has evolved into two separate subfamilies, with the split predating the origins of metazoans [188]. The UBF/HMGB family is present in all eukaryotic phyla, while the SOX/SRY family is present only in fungi and animals. The former shows only structure-specific interactions with DNA and RNA, while the latter family can make base-specific contacts [189]. HMGB1 has been reported to bind to multiple alternative conformations, including hemicatenated DNA loops (K_d_ = 0.2 × 10^−12^ M), DNA minicircles (1 × 10^−10^ M), and four-way junctions (1 × 10^−9^ M). The affinity for linear B-DNA is much lower (5 × 10^−5^ M) [190,191]. Binding to B-Z junctions [192] and to TRX [59] has also been reported. The interaction of HMGB family members with TRX is supported by the HMGB1 AF3 models (Figure 8H). Bending of DNA is evident in these folds, potentially altering the relationship between chromosomal segments and the alignment of enhancers with promoters.

### 2.6. Proteins That Modify Chromatin

#### 2.6.1. Polycomb Repressor Complexes

Chromatin remodelers also affect chromatin architecture, with PRC1 and PRC2 playing distinct roles. PRC1 promotes heterochromatin formation through the polyubiquitylation of H2A at lysine 119. The modification promotes the mono-, di-, and trimethylation of H3K27 by PRC2. The H3K27me3 mark then recruits additional PRC1 complexes to the site, creating a positive feedback loop [193].

**Figure 9:**
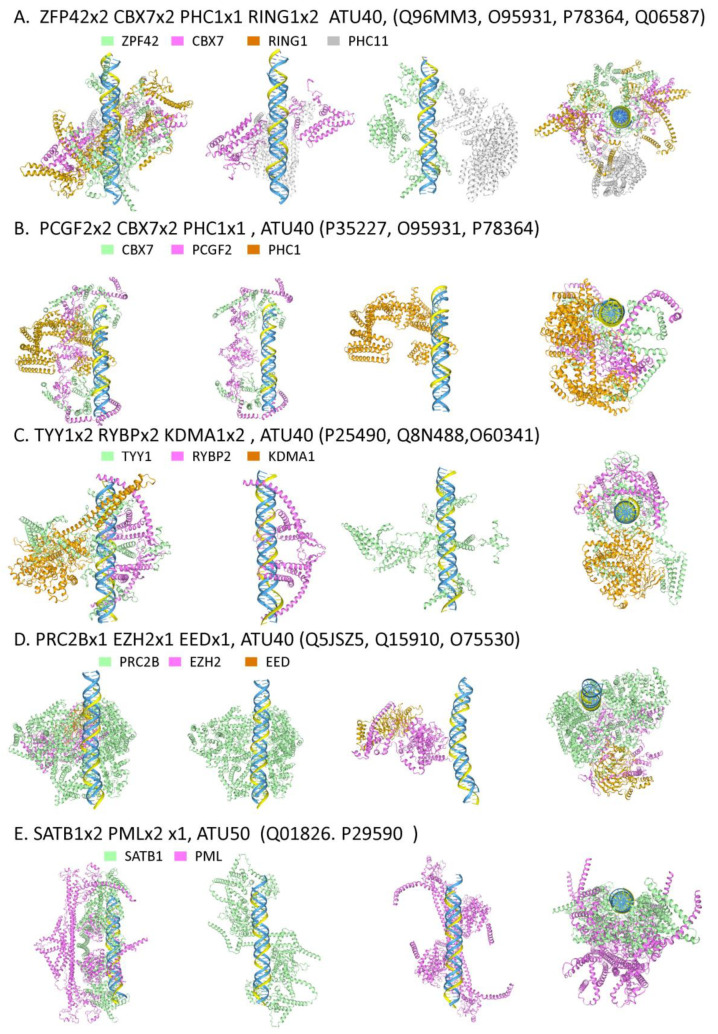
Epigenetic scaffolds bound to a parallel copolar d(AT)_50_rU_50_ triplex. (**A**–**C**) Different polycomb repressor complex 1 (PRC1) assemblies. (**D**) Core elements of the PRC2 complex. (**E**) Special AT-rich sequence-binding protein 1 (SATB1) and promyelocytic leukemia protein (PML). Each component of the complexes is uniquely colored as shown in the key. The different views show the position of each protein relative to the triplex. The TRX are shown as cartoons. DNA strands are colored blue and RNA strands yellow.

In AF3 models, both PRC1 and PRC2 dock to the TRX (Figure 9). The PRC1 complexes incorporate the ying-yang1 paralog zinc-finger protein 42 (ZFP42, also known as REX1) that is highly expressed in murine stem cells and represses expression of muERV-L/MERVL retrotransposons [194,195]. ZFP42 is also required in the pre-implantation embryo and extra-embryonic tissues for imprinted X chromosome inactivation (iXCI) of the paternal X chromosome. ZFP42 competes with YY1. The protein reduces the expression of the X-inactive specific transcript (Xist). This transcript can only be induced by YY1 from an unmethylated X allele.

AF3 modeling reveals that the ZFP42 PRC1 complex can dock to a TRX (Figure 9A). PRC1 activity is also associated with several other complexes [196,197]. In one of these, the unrelated Polycomb group RING finger protein 2 (PRCG2, known as Mel18) family member replaces ZFP42, thereby promoting the docking of CBX7 to TRXs (Figure 9B). Interestingly, in mammals, the ZFP42 paralog TYY1 does not appear to colocalize with PRC1 complexes, even though in AF3 models, it can replace ZFP42. It seems that following duplication, each TYY1 paralog has evolved under different selection pressures, consistent with Ohno’s views on how proteins acquire new functions [198,199]. In other AF3 models, YY1 docks to TRX in association with a distinct set of partners: RING1, YY1-binding protein (RYBP), and lysine-specific histone demethylase 1 (KDM1A, also known as LSD1) (Figure 9C). 

The DNA-binding specificity of PRC1 complexes remains an open question [197]. One accessory protein, Mel8, is also reported to bind a d(GACT) repeat that has a one-nucleotide spacer [200]. However, other PRC1 complexes do not appear to target specific sequences. While recognition of structures such as TRX provides one explanation, it has also been proposed that complex localization occurs through recognition of histone or DNA modifications. The two models are complementary and likely both correct. 

PRC2 also docks to TRX through a complex formed by five core components: enhancer of zeste homolog 2 (EZH2), embryonic ectoderm development protein (EED), suppressor of zeste 12 protein homolog (SUZ12), retinoblastoma-binding protein 4 (RBBP4), and RBBP7. The model shown in Figure 8D is without SUZ12 and RBBP4 or RBBP7. The primary contacts with the TRX are primarily mediated by proline-rich coiled-coil protein 2B (PRCB) with a contribution from EZH1. This and other models reveal that neither EED, RBB4, nor SUZ12 contacts DNA. EED contains a WD40 protein interaction domain, while SUZ12 interacts with PRC2 accessory proteins. RBBP proteins are also found in other complexes [197,201]. PRC2 can interact with other PRC1 and PRC2 complexes formed many megabases apart or on different chromosomes. The large assemblies generated are called polycomb bodies [202]. The diversity of complexes formed is enabled by various IDR folds seeded by alternative flipon structures.

#### 2.6.2. PML Bodies and SATB1

TRX can indirectly modulate the chromatin architecture and retrotransposition rates by sequestering chromatin-modifying enzymes into nuclear bodies. For example, the promyelocytic leukemia protein (PML) nuclear bodies encapsulate HP1, death domain-associated protein 6 (DAXX), and ATP-dependent X-linked helicase (ATRX) [203,204]. The formation of PML bodies is promoted by the special AT-rich sequence-binding protein 1 (SATB1), which was first noted to interact with adenosine-, cytosine-, and thymidine-rich sequences lacking guanosines. The protein binds to the DNA backbone, not to the bases, and docks to double-stranded, not single-stranded DNA. The bound sites are in DNA regions where high base-unpairing potential (BUR) [89] potentially enables the fold-back of a single-stranded DNA segment onto a duplex region to form a TRX, or the docking of an RNA to make an RDL.

In one study, in which stringent wash conditions were used, the majority of BURs colocalized with SATB1 in transcriptionally repressive lamina-associated domains. A subset of BURs bound by SATB1 (~18%) helped bridge open chromatin from different topological domains in a cell-type-specific manner [205]. Such strong, BUR-dependent interactions between widely separated DNA segments may partially explain why the expression levels of most genes remain essentially unchanged when TADs are destabilized by cohesin loss, and why chromatin compartmentalization was not affected [206,207]. Even loss of SATB1 in thymocytes did not affect the segregation of genes into A and B nuclear compartments, nor impact TAD formation, size, or number [205], suggesting that SATB1 did not induce the interaction between different regions, but rather formed a scaffold on BUR that other proteins bind.

The association with PML bodies provides further insight into the roles of SATB1. PML bodies orchestrate a diversity of biological outcomes, including the suppression of retroelement and viral gene expression, and the selective transcription of immune-related genes, such as those from the major histocompatibility complex [204,208]. AF3 models reveal that PML promotes the association of SATB1 with parallel copular TRXs (Figure 9E). Other models reveal that SATB1 can seed a set of complexes that include (SWI/SNF-related matrix-associated actin-dependent regulator of chromatin subfamily A member 5 (SMARCA5), REST corepressor 1 (RCOR), Nuclear autoantigen Sp-100 (SP100), Transcription factor PU.1 (SPI1), Transcriptional adapter 1 (TADA1), TADA2B, and Histone acetyltransferase KAT2A (GCN5).

#### 2.6.3. PIWI Chromatin Complexes

Compared to AGO, PIWI proteins have relaxed targeting rules, thought to help defend against rapidly evolving transposons [209]. Once bound, piRNAs promote the assembly of various complexes that limit retrotransposon spread. The engagement of TRX by PIWI proteins further exploits an ERE vulnerability, as the TRX-forming sequences are within conserved open reading frames. The overlap between flipons and codons makes evasion of this defense by mutation difficult, as loss-of-function variants will arise. The manner in which this vulnerability is exploited varies by species [210,211].

**Figure 10:**
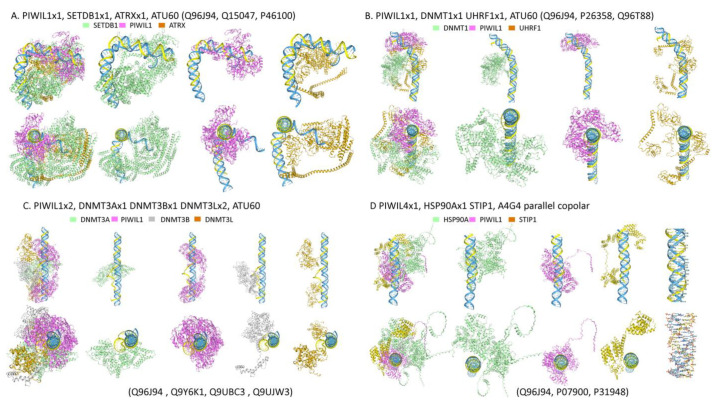
Different PIWI-seeded complexes. PIWIL1 bound to a parallel copolar d(AT)_60_rU_60_ triplex. (**A**–**C**) and PIWIL4 bound to A4G4 repeat triplex (**D**). (**A**) Docking or the histone-lysine N-methyltransferase SETDB1 is assisted by the transcriptional regulator ATRX. (**B**) Docking of the DNA (cytosine-5)-methyltransferase 1, DNMT1, is scaffolded by the E3 ubiquitin-protein ligase UHRF1. (**C**) PIWIL1 also promotes docking of the DNA (cytosine-5)-methyltransferase 1 DNMT3A complex to a triplex. (**D**) The binding of heat shock protein 90-alpha (HSP90A) is mediated by PIWIL4 and the stress-induced-phosphoprotein 1 (STIP1). In the panels, each component of the complexes is uniquely colored as shown in the key. The different views show the position of each protein relative to the TRX. The TRXs are shown as cartoons, with bases numbered by strand. DNA strands are colored blue and RNA strands yellow. DNA bases are further denoted by “d”. TRXs are also drawn as ball-and-stick representations in standard colors. Hydrogen bonds are indicated by dotted lines.

In humans, both suppressive histone marks and DNA methylation appear to be promoted by the PIWI-related PIWIL1 (HIWI, encoded by *PIWIL1*) proteins [212,213]. However, the proteins necessary for these modifications contain many IDRs and do not fold well in AF3 when tested individually. AF3 models were used to evaluate the folding of these proteins in the presence of PIWI proteins, and a copolar TRX composed of U_60_ RNA docked to a d(AT)_60_ duplex. Under these conditions, the ATP-dependent helicase ATRX and the histone-lysine N-methyltransferase SETB1, which catalyzes H3K9me3, yielded a well-formed complex. In this structure, PIWIL1 induced a profound bend in the TRX, similar to that induced by nucleosome binding. The bend likely enables modification of the neighboring histones by SETB1 (Figure 10A) [214]. In another model, PIWIL1 facilitated the binding of the E3 ubiquitin-protein ligase UHRF1 and the docking of the DNA methyltransferase I (DNMT1) (Figure 10B). The complex links histone modification to the maintenance of DNA methylation during DNA repair and cell division [215]. PIWIL1 also localizes the de novo DNMT3 methylation three-subunit complex to the TRX. The DNMT3L protein binds unmethylated histone H3 lysine 4 to recruit or activate the DNMT3 catalytic subunits (Figure 10C). The complex suppresses EREs during embryogenesis and establishes the imprinting of maternal genes [216].

PIWI in both flies and plants directs the assembly of complexes that suppress transposon activation during heat shock responses [217,218,219]. These complexes are formed with heat shock protein 90-alpha (HSP90A) and the stress-induced-phosphoprotein 1 (STIP1), which acts as a co-chaperone for HSP90A. The complexes suppress the phenotypic variations in both flies and plants that arise from the activation of ectopic ERE enhancers and promoters. A similar mechanism is likely active in humans. In AF3 models, human PIWIL1 promotes the docking of HSP90A and STIP1 to the A4G4 repeat parallel, coplanar TRX (Figure 10D). The question of whether such interactions are directed by piRNAs or other types of small ncRNAs has been examined in tumors, where PIWIL4 levels are elevated. In acute myeloid leukemia cells, the RNase activity of PIWIL4 is essential, and protects against activation of the ataxia-telangiectasia mutated (ATM) and ATR and RAD3-related (ATR) repair pathways, as well as preventing R-loop formation. Only 6% of the RNAs bound by PIWIL4 map to known piRNAs. The others engage cancer-associated protein-coding RNAs [136], suggesting that PIWIL4 generates these fragments de novo from DRHs, after localization to RDLs, via its RNase H activity. Such a mechanism then allows PIWIL4 to suppress active EREs and their transcripts. The possibility is supported by a meta-analysis of four cancer types. Mortality was lower among patients with higher levels of PIWIL4 expression, assessed by either mRNA or protein measurements, suggesting that the pathway confers a differentiated and more benign tumor phenotype [220].

## 3. Discussion

### The Role of TRX-Forming Flipons in Cellular Transactions

The AF3 models reveal that TRXs of various types induce well-folded protein complexes, even with proteins that have many IDRs. Like other flipons, TRXs are formed mainly by actively transcribed repeat sequences present in open regions of chromatin. The TRX modelled here incorporates RNA as the third strand, either produced in cis by actively transcribed genes or in trans by small and large noncoding RNAs. While the TRXs require base-specific interactions, the complexes they seed do not. The proteins appear to accommodate TRX with different sequence compositions and strand orientations. The various proteins bound by TRX substrates fold adaptively. Their intrinsically disordered domains and the flexibility of their arginine and lysine side chains enable a variety of interactions. The TRX sequences also facilitate the formation of complexes with varying degrees of DNA bending. The assemblies can facilitate local tethering of DNA to the nuclear envelope or alter chromatin architecture by forming or breaking contacts between sites that are typically distant.

**Figure 11:**
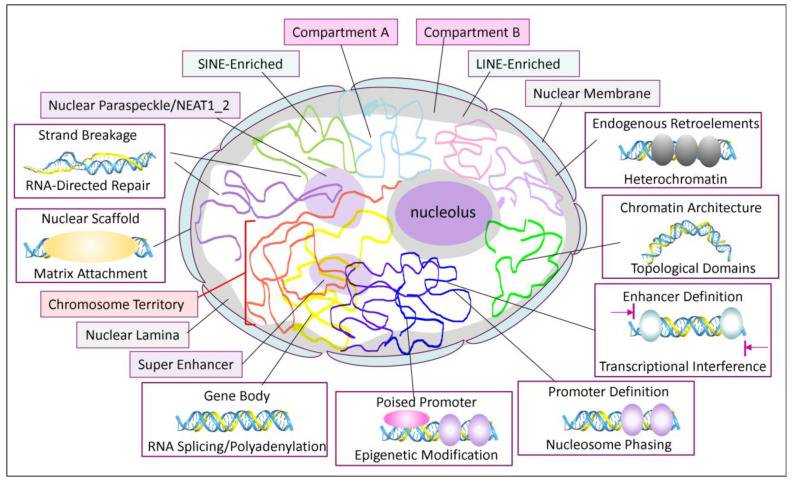
TRX-mediated transactions. The white boxes describe the different roles of triplex-binding proteins elaborated in the text. Colored boxes highlight various architectural features within the nucleus. Ellipses indicate protein or protein complexes, with their role described in the same box. The arrows indicate the direction of RNA polymerase movement. The vertical bars indicate a block.

An issue raised by the AF3 models concerns the relative contribution of apolar and copolar TRXs to cellular biology. In vitro studies reveal that, in general, apolar TRXs are more stable than copolar ones [104,105]. In some cases, the answer is not so clear. It is generally assumed that RDLs formed at the site of transcription form R-loops rather than copolar TRXs. There is evidence, both experimental and from MD simulations, that this may not always be the case [94]. In three-stranded recombination structures, the matching strands are copolar, as is the case for the TRX formed by stretched ssDNA [103,221]. In these complexes, rapid exchanges of short nucleotide segments occur during the homology search [222]. A similar action may allow AGO and PIWI-bound RNAs to invade duplex DNA or to dock within R-loops. The interactions of other proteins with RDLs may also promote the formation of TRX rather than R-loops. The binding of TP53 and RAD51 to RDLs would help protect the genome by preventing the formation of R-loops (Figure 4). Other RDLs may also be resolved by promoting TRX formation. By pausing transcription, the TRX may provide time to assemble a particular complex at that site. In other cases, the formation of more stable apolar TRXs by trans-acting RNAs may be better suited to phasing nucleosomes (Figure 11).

AF3 modeling reveals that the heat shock-induced complex docks to RDLs, preferentially to copolar, parallel-strand TRXs (Figure 10D). These complexes help buffer phenotypes against environmental stresses, consistent with the concept of canalization proposed by Waddington [223]. Indeed, the interaction of PIWI with RDLs, in concert with HSP90A and STIP1, suppresses phenotypic variations in both flies and plants. These effects extend beyond the repression of retrotransposons [217,218,219]. The complexes formed potentially mask the ectopic promoters, enhancers, and flipons exapted early on from EREs, but now employed to drive developmental programs. By targeting RDLs formed during heat shock, PIWI and HSP90A halt reactivation of stem cell pathways that otherwise result in sports. In fly embryos, RDLs are enriched with LINEs and LTRs, while in or zebrafish zygotes, RDLs are formed by microsatellite repeats and LTRs, such as Copia or Gypsy [224,225,226]. Those retrotransposons activated by environmental stresses instead act as gene drives, spreading their adaptation through the germline [227,228].

More generally, proteins engaged by TRXs can seed many different complexes in different cells at different times. Contrary to the prevailing static B-DNA models, many of the interactions that regulate transcription are transient [3], and depend on IDRs. Their effects will depend on the local concentration of each potential partner and on the modifications that alter IDR folding, stability, and cellular localization. Each interaction seeds the next. The complexes form dynamically, rather than through a pre-specified lock-and-key mechanism. The outcome is biased toward components with the slowest off-rates. The specific assembly created will depend on the cell type, the stage of the cell cycle, and the state of differentiation. The process is dynamic, with complexes constantly culled as the context changes their availability and their modifications. The arrangement generates immense phenotypic variability, with canalization essential for maintaining current fitness.

Noncoding RNAs are central to these diverse outcomes. They protect against EREs while also enabling rapid adaptation. Their central role in metazoan evolution has been both advocated by Britten and Davidson [229], and rejected in favor of a protein-centric scheme [230]. A highly significant difference between these two opposing world views was highlighted by Dickson and Robertson [231]. While an RNA sequence of 17 nucleotides specifies a unique location in the human genome, a protein regulator requires many more nucleotides to encode the necessary information. For example, the lac repressor of *E. coli* requires 1080 base pairs. Subsequently, evidence for many roles of noncoding RNAs in the cell has accumulated, fulfilling many aspects of the schema proposed by the noncanonical [231].

The new results reaffirm the idea that prebiotic entities exploited the ability of nucleic acids to self-amplify and to fold alternative structures. From this early tinkering, a non-overlapping genetic code evolved, enabling the protein-based chemistry on which modern cells are built [2]. Proteins facilitated more efficient transcription and replication of the early tinkers. Later, proteins enabled rapid triage of defective RNAs arising from splicing or pre-mRNA processing errors. They improved protein quality by eliminating RNAs with premature termination codons. They tracked RNA processing by adding N6-methyladenosine tags during transcription and by monitoring their removal as introns were excised and messages were polyadenylated [232,233,234]. The RNA components provided the essential complexity, with multiple different transcripts produced from the same gene, yet only a limited number of protein variants [235]. Many of the ncRNAs are likely an incidental byproduct of how genomes evolve. The new variants appear without replacing past adaptations. In contrast, changes to coding sequences lead to loss of the previous protein product of a gene. Despite the emergence of the protein world, nucleic acids remain indispensable to many outcomes. The ribosome still uses RNA rather than proteins to catalyze peptide bond formation. Other RNAs retain catalytic activity [236,237]. 

The sequence-specific readout of information from genomes by proteins was a later innovation. Proteins were robust and better suited to solving challenges in complex environments. This outcome is exemplified by proteins that fold stably to optimize the chemistry they perform, even under extreme conditions. Less-obvious instances involve proteins with IDRs. These regions can fold in multiple ways, depending on the state of the cell. They can read out nucleic acid structure to seed different complexes. The protein assemblies formed vary with context, and differ from the low-energy versions captured by current crystal structures. Only now are cryo-EM structures allowing us to explore the world beyond B-DNA-specific interactions.

Both protein and RNA modifications bias the assembly of complexes. Alternative reading frames, splicing, and targeted elimination of ncRNAs further canalize the protein folds [223], as do the flipon structures formed. Many of the ncRNAs that direct these outcomes originate from ERE, producing redundancy and increasing the robustness of the responses they coordinate. Many ncRNA variants can arise rapidly. These transcripts can add or delete sequence-specific protein-docking sites, induce different IDR folds, and modulate flipon structure [238]. The novel assemblies they seed then compete with past successful cellular adaptations, each subject to natural selection [239].

## 4. Methods

PATO (V1.01) was used to identify triplex forming sequences using the default parameters [92].

AF3 (https://alphafoldserver.com/, accessed on 16 January 2026) is not explicitly trained to recognize TRX nor their interactions with proteins. However, large neural networks often contain sub-networks that, when properly initialized, can perform well on specific tasks [240]. In this application, complexes are anchored by TRXs formed with an RNA third strand and a DNA duplex, representing low-energy minima in the folding landscape. A further challenge to AF3 is the folding of intrinsically disordered regions (IDRs). Under certain conditions, AF3 can identify folded structures for a subset of IDRs (conditional folding), rather than producing barbed wire, structural overlaps, or hallucinations [241,242,243]. Potentially, AF3 can correctly identify structures that are stable but differ from the lowest-energy forms captured by crystals. In particular, alternative flipon structures that represent distinct energy minima in the folding landscape can nucleate different IDR folds.

Consistent with the known AF3 limitations, it was found that a subset of putative triplex-forming sequences would not fold into a three-stranded structure and that steric clashes would arise with structural overlaps of copolar chains. In a number of cases, the incorporation of non-canonical triplets would overcome this limitation by prompting the correct fold. The analysis here uses the subset of well-folded triplexes with canonical triplets (Figure 2). By selecting a particular AF3 seed and a set of ion conditions, it was possible to explore the longer triplexes formed by extended repeats. These triplexes have not been experimentally analyzed previously.

For protein interactions involving AGO, PIWI, and REL-domain families, the same triplet (G-rich, parallel, apolar) was used in all models. This approach enables the direct comparison of these models and provides evidence that the AF3 folds are replicable and robust. For larger complexes, a d(A:T)*rU was used to form TRXs. Empirically, it was found that AF3 can be nudged by setting input conditions and specifying the model seed, enabling exploration of the interactions between TRX and proteins. All the models presented use the same seed and differ only in the number of zinc ions included. This approach has successfully mapped the interaction between the ADAR Zβ domain and G-quadruplexes. This prediction has subsequently been confirmed first by Molecular Dynamics simulation, then by NMR [244,245]. Other interactions have been verified using this approach but are not yet published. In the case of zinc-finger proteins, experimental support for a subset of these models was available from previously published work [158].

During this work, it was found that the AF3 default scoring of these models did not always assign high scores to the well-validated interaction between Zα and Z-DNA. Instead, a better indication of their significance was provided by manual inspection of the models produced. The results presented here fold entire proteins, either singly or in association with other protein interactors. Many of these proteins contain large IDRs that fold in the presence of a TRX. Different TRX-forming sequences were tested to determine those that induced fully folded proteins. Models with many well-oriented bonding schemes and without steric clashes were selected for further evaluation.

It was found that in the presence of a TRX, many proteins with extensive IDRs folded completely, whereas under many other conditions, they did not. The results suggest that TRXs can act as chaperones to promote the local assembly of the cellular machinery required for epigenetic modification of chromatin architecture. The PDB files corresponding to the figures are provided in Supplementary Data File S3. The number of ions used in the model, plus the seed, is specified in the file title.

These models provide evidence for the proposed interactions and motivate further validation through Molecular Dynamics simulations. Targeted experimental follow-ups include (1) single-molecule FRET to validate IDR folding upon TRX binding; (2) X-ray crystallography or cryo-EM to resolve the atomic structure of high-priority complexes; and (3) in vitro binding assays to quantify TRX-protein affinity and test the specificity of predicted interfaces; (4) targeted mutagenesis of residues mediating interactions between proteins and triplexes, both in vitro and in vivo. Overall, the models provide evidence for a central role of triplex-forming flipons in regulating cellular chromatin states and context-specific transcriptomes.

## 5. Conclusions

ncRNAs also remain essential to rapid adaptation in many ways. By setting open chromatin regions, they allow TFs to extract different sets of information from the genome. Flipons modulate the timing and reinitiation of these processes by cycling between different structures to seed cell-specific versions of the chromaverse. However, these roles for ncRNAs were overlooked because they are most often encoded by repeat elements, which constitute the majority of the genome. These repeats were viewed negatively as error-prone and the primary cause of genomic instability. Their high frequency also rendered them uninformative. However, as shown here and in earlier papers, the flipon subset is highly informative.

By switching conformations, flipons flag active regions of the genome. The simple structures they form induce folding of intrinsically disordered proteins and seed the context-dependent condensates that generate complexity [4]. Through these processes, each flipon fold has a different impact on phenotype. Flipon structural transitions also promote the turnover of the bound complexes, enabling updates based on the cell’s current state. Resets and interactions between different flipon types nowadays depend on both proteins and ncRNAs [137]. These systems are highly adaptive. Many of the complexes they seed help protect the cell against EREs. They coordinate responses by forming nuclear and cytoplasmic scaffolds. They regulate the real-time readout of genetic information through the just-in-time compilation of genetic programs [57]. As such, flipons are subject to natural selection through the innovations they enable.

Within this framework, flipons connect the early tinkers with the lifeforms that we see today [2]. ADAR1 is perhaps the simplest example of this paradigm, in which structure begets specificity. The interactions of ADAR1 domains with Z-DNA, G-quadruplexes, and dsRNA are structure-specific yet yield sequence-specific recoding of RNAs [244,245,246]. This enzyme protects cells against ERE and produces protein variants that are not explicitly encoded by the genome [246]. Other structure-based mechanisms, implemented with flipons and noncoding RNAs and not genetically encoded, produce phenotypic diversity. Over time, a variety of ncRNAs evolve that regulate flipon conformation and the transcription, processing, stability, and turnover of other RNAs [247]. As a result, each cell in the body is likely unique, as each colors its chromaverse differently. Well-adapted cells undergo positive selection, and a subset of each clone will come to populate healthy tissues. The challenge now is to therapeutically reprogram the clades of dysfunctional cells that cause disease.

## Data Availability

Triplexes predicted for retroelements using PATO (Version 1.01) are given in Supplementary Data File S1 and for lncRNAs in Supplementary Data File S2. PDB files for the structures shown in the Figures are supplied in Supplementary Data File S3.

## Supplementary Materials

The following supporting information can be downloaded at: https://www.mdpi.com/article/10.3390/ijms27031482/s1.
